# Evaluating the Barcelona Music Reward Questionnaire and semantic differential profile scale for adults with major depressive disorder and suicidal ideation

**DOI:** 10.1371/journal.pmen.0000361

**Published:** 2025-09-05

**Authors:** Melissa Tan, Sakina J. Rizvi, Corene Hurt-Thaut, Michael H. Thaut

**Affiliations:** 1 Music and Health Sciences Research Collaboratory, Faculty of Music, University of Toronto, Toronto, Ontario, Canada; 2 ASR Suicide and Depression Studies Unit, St. Michael’s Hospital, Toronto, Ontario, Canada; 3 Department of Psychiatry, University of Toronto, Toronto, Ontario, Canada; 4 Faculty of Medicine, University of Toronto, Toronto, Ontario, Canada; University of Canterbury, NEW ZEALAND

## Abstract

Reward processing abnormalities in individuals with Major Depressive Disorder (MDD) alter motivation and hedonic responses, with greater impairments in those at risk of suicide. Preferred music is typically perceived as a rewarding and pleasurable experience that can influence motivation and emotion. Given its influential nature, evaluating how individuals with MDD and suicidal thoughts and behaviours respond to music could serve as a novel behavioural marker to indicate suicidality. This study aimed to evaluate reward response to music measured through the Barcelona Music Reward Questionnaire (BMRQ) and a semantic differential profile scale to investigate music reward and self-perception. Data were collected through the CAN-BIND 5 initiative at St. Michael’s Hospital, Toronto. Adults aged 18–68 years participated in this cross-sectional observational study. Participants included individuals with MDD with suicidal ideation and no attempt (SI/NA; n = 19), MDD with suicidal ideation and lifetime attempt (SI/LA; n = 14), MDD with no suicidal ideation and lifetime attempt (NSI/LA; n = 13), and healthy controls (HC; n = 28). Participants completed the BMRQ online within 48-hours of their baseline visit. During the in-person visit, they completed a 29-item semantic differential profile scale before and after listening to a preferred piece of music. BMRQ results showed no significant differences across groups for each factor and for overall scores. A two-way ANOVA with the semantic differential profile scale revealed a significant interaction effect between participant group and time points, *F*(3, 224) = 4.32, **p* *= .006, η^2^ = 0.05. Post-hoc analysis using Tukey’s HSD test revealed the HC consistently had significantly higher semantic profile scores than all patient groups, both before and after listening to music (all **p* *< .001). Additionally, all patient groups exhibited a significant change in semantic differential profile scores from before to after music listening (all **p* *< .001). These findings highlight that music engagement was experienced similarly across groups and that the BMRQ was not sensitive enough to detect differences. However, the semantic differential profile scale was sensitive enough to capture shifts in self-perception from before to after listening to music, indicating its potential as a behavioural indicator for suicidality.

## Introduction

Suicide represents a significant public health issue worldwide, claiming over 720,000 lives each year. It ranks as the fourth leading cause of death among individuals aged 15–29 [1]. Those diagnosed with Major Depressive Disorder (MDD) face an elevated risk of engaging in suicidal behaviours [2]. Given the global prevalence of suicide and the associated risks for individuals with MDD highlight the critical necessity for practical, accessible, and sensitive tools to understand suicidality. A promising avenue for identifying such behavioural markers lies in the domain of reward processing, which is often disrupted in individuals with MDD.

Reward processing differs in individuals with Major Depressive Disorder (MDD), especially when considering the presence of suicidal thoughts and behaviours. Reward processing refers to the way individuals respond to and engage with specific stimuli that can motivate behaviour, predict future rewards, and influence affective states, thereby shaping specific behavioural responses [3]. The reward processing abnormalities in individuals with MDD alter motivation and hedonic responses, with more pronounced impairments observed in those at risk of suicide [4]. Reduced reactivity to reward stimuli in areas of desire for reward, anticipation, motivation, pleasure, and reward learning can contribute to the presence of anhedonia [5]. Anhedonia, characterised by reduced or complete absence of the ability to experience pleasure, is a hallmark feature of MDD [6]. Neuroimaging studies analysing the neural correlates of anhedonia have shown decreased activity of the ventral striatum and nucleus accumbens, and increased activity within the ventromedial prefrontal cortex and the orbitofrontal cortex [7].

Due to the reward processing differences apparent in individuals with MDD and suicidal thoughts and behaviours, it is essential to explore methods that evaluate reward and pleasure responses. Current assessments for reward processing include self-report measures that rely on experiences over a specific period of time such as the Snaith-Hamilton Pleasure Scale (SHAPS) and Temporal Experience of Pleasure Scale (TEPS), behavioural tasks primarily using monetary incentives, and physiological and neuroimaging measures [8]. However, there is a critical need to identify reliable behavioural markers for suicidality to improve early detection and intervention. Given the multifaceted and complex nature of reward processing, assessing reward through the lens of something more social or intrinsic, like music, may provide another dimension of how rewards are processed.

Music inherently engages and motivates individuals, eliciting profound emotional responses due to its processing within the brain’s reward system. Listening to pleasurable music activates reward-related regions, including the ventral striatum and nucleus accumbens [9–14]. Blood and Zatorre [9] found that intense pleasure responses, such as “chills,” correlate with physiological changes and activity in brain regions involved in reward, emotion, and arousal, including the anterior cingulate cortex and insula. Koelsch et al. [11] showed that pleasant music activates Heschl’s gyrus, the anterior superior insula, and the left inferior frontal gyrus, with these activations increasing over time. Suzuki et al. [14] and Trost et al. [15] identified specific brain regions associated with emotion-specific processing in music, such as the striatum and orbitofrontal cortex, depending on the emotional valence and arousal level of the music. These neuroimaging findings highlight the complex neural processes involved in emotional responses to music, making it particularly valuable for understanding reward-related deficits in individuals with MDD and suicidality.

In addition to music’s role in emotional responses and reward processing, it may also influence and shape how individuals perceive themselves. Self-perception refers to how individuals view and evaluate their traits, emotions, and identity, contributing to their sense of self and engagement with life experiences [16]. In the context of suicidal ideation and MDD, self-perception is often negatively skewed, with individuals experiencing persistent feelings of worthlessness, hopelessness, and lack of purpose [1,2]. These perceptions are central to suicidality and may contribute to increased risk. Music, specifically preferred music listening, may offer a way to understand subtle shifts in self-perception as it can evoke personal memories, support emotional expression, and foster a sense of connection [17].

Due to its influence on emotional and motivational states, music may serve as a novel tool for assessing music reward experiences and self-perception in individuals with depression and suicidality. This study explores how music engagement and perception of self before and after listening to music is experienced with individuals with varying levels of suicidal ideation with and without an attempt history compared to healthy controls, utilising the Barcelona Music Reward Questionnaire (BMRQ) and a semantic differential profile scale.

The BMRQ was developed by Mas-Herrero et al. [18] and is a psychometric tool to assess how individuals engage with music. The BMRQ explores different facets of musical reward experiences using a 20-item questionnaire divided into five main factors. These factors are defined as follows: musical seeking (the drive to actively seek music), emotion evocation (using music to elicit emotional responses), mood regulation (using music to influence mood), social reward (connecting with others through music), and sensory-motor (physical reactions like dancing or tapping along) [18]. There is high reliability and validity across different languages and cultural contexts, including English, Spanish, and French, with significant individual differences in musical reward experiences linked to socio-demographic factors and general sensitivity to reward and hedonic experiences [19]. The self-reported nature of the BMRQ provides insights into how individuals experience and engage with music.

The semantic differential scale is a psychological tool that creates semantic space to capture the nuanced attitude of self using a series of bipolar constructs (e.g., annoyed – pleased, sleepy – alert, sad – happy) [20]. The flexibility of the scale can provide the intensity and direction of attitudes towards self, which can provide valuable insights into an individual’s psychological state [20]. In the present study, an in-house version of the semantic differential profile scale was developed by a research group affiliated with the Music and Health Sciences Research Collaboratory (MaHRC) at the University of Toronto. This was specifically designed to understand the characteristics of this clinical population. The rationale for developing an in-house version of the semantic differential profile scale was to capture subtle, immediate shifts in self-perception that may or may not be detected by other measures. This version was adapted to reflect constructs relevant to emotional and physical attitudes in this clinical population. This scale assessed attitude of self and if there were immediate changes in their self-perceptions after a music experience (i.e., music listening).

Due to the involvement of music in emotion and reward processing, this study aimed to further investigate how music is experienced by adults with varying levels of suicidal ideation, with and without an attempt history. By utilising the BMRQ to understand overall music reward experiences and a semantic differential profile scale to assess changes in self-perception before and after listening to music, the study aimed to determine if response to music can serve as behavioural indicators of suicidality.

## Methodology

### Data source overview

The Canadian Biomarker Integration Network in Depression (CAN-BIND) is a research initiative has focused on understanding and improving the treatment of depression using biomarkers [21]. The goal is to integrate biological, psychological, and social data to provide more effective treatments for individuals with depression. CAN-BIND 5 is an arm of the research initiative which took place at St. Michael’s Hospital, Toronto, and examined the biomarkers of suicidality [22]. This study was approved by the Unity Health Toronto Research Ethics Board (REB#15–091). This study was conducted in accordance with the ethical standards of the institutional research committee, the Tri-Council Policy Statement: Ethical Conduct for Research Involving Humans, and the Declaration of Helsinki. The present research project uses data collected from this study to further understand how individuals with MDD with and without a history of suicidal behaviour experience music and their attitudes towards self before and after listening to music.

### Participant recruitment

Adults aged 18–68 years were enrolled from the summer of 2017 to the summer of 2024. They were recruited through the Department of Psychiatry at St. Michael’s Hospital (Toronto, Ontario), an existing database where consent to be contacted for future studies was given, and advertisements in the community and on Facebook.

Four participant sample groups were enrolled in the study:

- Group 1: MDD patients with suicidal ideation and no attempt (SI/NA)
- Group 2: MDD patients with suicidal ideation and lifetime attempt (SI/LA)
- Group 3: MDD patients with no suicidal ideations and lifetime attempt (NSI/LA)
- Group 4: Healthy controls (HC)

All participants with MDD had a clinical diagnosis meeting the DSM-V criteria of MDD confirmed through the Mini-International Neuropsychiatric Interview (M.I.N.I.); were between the ages of 18 and 70 years, scored ≥ 14 on the Hamilton Depression Rating Scale (HAMD-17); and could give informed consent. For SI/NA and SI/LA groups only, participants had to score ≥ 2 on the HAMD-17 item 3, indicating suicidal ideation in the past week. For SI/LA and NSI/LA groups only, participants had a positive history of a lifetime suicide attempt, determined by the Columbia Suicide Severity Rating Scale (C-SSRS).

Participants were excluded if they were pregnant; had a medical condition requiring immediate investigation or treatment; a recent (≤ 6 months) or current history of drug abuse or dependence other than caffeine or nicotine; and a lifetime history of psychosis, Bipolar I or Bipolar II. Participants who were taking a daily sedative hypnotic, atypical antipsychotic, or stimulant were on a stable dose for at least 4 weeks prior to participation.

Healthy controls were between the ages 18 and 70 years; had no lifetime history of Axis I disorders; had no lifetime history of antisocial personality disorder or borderline personality disorder; had no history of antidepressant use; and had no treatment for an acute or ongoing medical condition.

### Study design

This study utilised a cross-sectional observational study design. Upon providing written informed consent, participants were emailed the Barcelona Music Reward Questionnaire (BMRQ) and had an in-person baseline visit where music listening and the semantic differential profile scale was completed.

### Data collection procedures

All participants who met inclusion criteria provided written informed consent prior to enrolment. Following written informed consent, the participant’s eligibility was confirmed with a screening visit conducted remotely over Zoom or in person. If eligibility was met, the participant continued with a baseline in-person study visit. At the baseline visit, participants provided demographic information and completed the M.I.N.I., HAMD-17, and Columbia Suicide Rating Scale (C-SSRS) to confirm eligibility.

Participants were sent the Barcelona Music Reward Questionnaire (BMRQ) via email and asked to complete this within 48 hours of their baseline visit. If incomplete, the BMRQ was completed at the baseline visit. This self-report questionnaire measured five facets of music experience, including how individuals experience reward associated with music [18].

At the in-person visit, participants were instructed to identify a preferred piece of music between 5 and 7 minutes in length to listen to. If necessary, verbal cues for selecting a piece of music were used to assist the participant. The chosen piece was then queued up on YouTube for the participant to listen to through headphones. Visuals from the YouTube video were hidden from the participants. The participant completed a 29-item semantic differential profile scale, developed by a research group associated with the Music and Health science research Centre (MaHRC) at the University of Toronto, assessing their emotional and physical state before and after listening to their preferred piece of music (see Table 1). While the scale has not undergone formal psychometric validation, it was designed to align closely with the constructs of interest.

**Table 1:** Semantic differential profile rating constructs.

Constructs
Annoyed – Pleased
Melancholic – Contented
Lonely – Cared for
Timid – Bold
Dull – Bright
Indifferent – Caring
Sleepy – Alert
Gloomy – Cheerful
Despairing – Hopeful
Unfulfilled – Satisfied
Sad – Happy
Weak – Strong
Detached – Connected
Awful – Nice
Blue – Delighted
Anxious – Calm
Dispirited – Lifted
Bitter – Joyful
Upset – Composed
Unsentimental – Nostalgic
Bored – Excited
Regretful – Defiant
Heavy – Light
Close-minded – Broad-minded
Worn out – Active
Overwhelmed – In control
Cold – Warm
Irritated – Tranquil
Distressed – Safe

### Statistical analysis

To assess the outcomes of the BMRQ, the raw scores were transformed into factor scores via an Excel file provided by Cardona et al. [23], to accurately reflect the underlying constructs identified in the original study to reduce measurement error [18]. A one-way ANOVA was conducted to compare the factor mean scores across groups.

To evaluate the semantic differential scale profile rating, overall mean scores derived from the scale for each group were calculated. A two-way ANOVA was conducted to assess main effects of participant group and time points (before and after listening to music), as well as their interaction, on the semantic differential profile scores.

All statistical analyses were done in R version 4.4.1.

## Results

### Participant description

Seventy-four participants participated in this study, with nineteen participants with SI/NA, fourteen with SI/LA, thirteen with NSI/LA, and twenty-eight healthy controls. One participant in the SI/NA group and two in the SI/LA group did not complete the semantic differential profile scale. One participant in the NSI/LA group did not complete the BMRQ. Participant characteristics are described in Table 2, including those who did not complete both outcome measures.

**Table 2:** Characteristics of study participants.

	Group 1: SI/NA^a^ n = 19	Group 2: SI/LA n = 14	Group 3: NSI/LA n = 13	Healthy Controls n = 28
Characteristic				
Age, y *M(SD), range*	39 (12.07), 19-65	32.14 (13.20), 18-60	37.23 (13.22), 18-59	40.04 (15.37), 18-68
Sex (male: female)	10:9	5:9	4:9	13:15
Handedness (n)				
Right	15	13	12	25
Left	2	1		2
Ambidextrous	1		1	1
Unknown	1			
Number of past suicide attempts (n)^b^				
0	19			
1		6	5	
≥ 2		8	8	
Psychotropic medication use at baseline^c^	12	10	10	
Antidepressants^d^				
SSRI	6	6	2	
SNRI	4	6	7	
NDRI	3	4	5	
SARI		2	3	
TCA	1	1		
SMS	1	1		
NaSSA			1	
Antipsychotics	3	5		
Antiepileptic	2	1		
Anxiolytics and Hypnotics	7	5		
Stimulants	5	1		
Cannabinoid	2			
HAMD^e^ *M(SD)*	21.2 (4.8)	21.1 (4.2)	19.9 (4.2)	

*Note.*

^a^Patient Groups: SI/NA: suicidal ideation and no attempt, SI/LA: suicidal ideation and lifetime attempt, NSI/LA: no suicidal ideations and lifetime attempt;

^b^Number of past suicide attempts in lifetime;

^c^Stable medication of at least four weeks;

^d^Antidepressant abbreviations: selective serotonin reuptake inhibitor (SSRI), serotonin-norepinephrine reuptake inhibitor (SNRI), norepinephrine-dopamine reuptake inhibitor (NDRI), serotonin antagonist and reuptake inhibitor (SARI), tricyclic antidepressant (TCA), serotonin modulator and stimulator (SMS), noradrenergic and specific serotonergic antidepressant (NaSSA);

^e^Hamilton Depression Rating Scale (HAMD) ranges: Normal = 0–7, Mild Depression = 8–13, Moderate Depression = 14–18, Severe Depression = 19–22, Very Severe Depression = ≥23.

### Barcelona Music Reward Questionnaire

**Fig 1:**
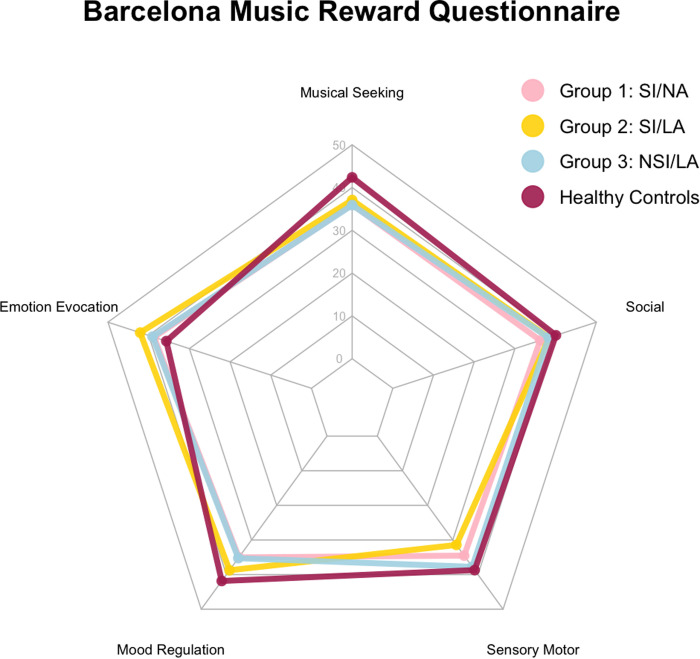
Radar graph representation of the mean factor scores of each group. *Note.* No statistically significant differences were found among participant groups.

The BMRQ resulted in the following overall music reward mean scores: SI/NA had a mean of 40.6 (*SD *= 15.9), SI/LA had a mean of 43.0 (*SD *= 12.3), NSI/LA had a mean of 42 (*SD *= 11.6), and healthy controls had a mean of 46.3 (*SD *= 10.3). Descriptive statistics were calculated for each BMRQ factor across the different participant groups are presented in Table 3 and a comparison of mean factor scores is illustrated in Fig 1.

**Table 3:** Descriptive statistics for each BMRQ factor.

	Group 1: SI/NA n = 19	Group 2: SI/LA n = 14	Group 3: NSI/LA n = 12	Healthy Controls n = 28
Factor *M(SD)*				
Musical Seeking	43.2 (12.6)	44.5 (13.2)	42.1 (14.0)	50.9 (8.2)
Emotion Evocation	46.4 (12.1)	50.5 (9.4)	46.8 (10.4)	42.8 (13.1)
Mood Regulation	42.1 (15.3)	46.5 (10.7)	41.8 (8.3)	50.2 (8.8)
Sensory Motor	41.4 (14.5)	37.7 (11.8)	45.5 (12.5)	46.4 (12.0)
Social Reward	43.5 (13.7)	46.1 (11.6)	45.8 (12.6)	48.1 (10.2)
Overall Music Reward	40.6 (15.9)	43 (12.3)	42 (11.6)	46.3 (10.3)

*Note.* Patient Groups: SI/NA: suicidal ideation and no attempt, SI/LA: suicidal ideation and lifetime attempt, NSI/LA: no suicidal ideations and lifetime attempt.

A one-way ANOVA compared patient groups and healthy controls for the five BMRQ factors and overall music reward.

The results indicated no significant difference for all factors: musical seeking, *F*(3,69) = 2.59, **p* *= .06, η^2^ = 0.10; emotion evocation, *F*(3,69) = 1.40, **p* *= .25, η^2^* *= 0.57; mood regulation, *F*(3,69) = 2.71, **p* *= .05, η^2^ = 0.11; sensory motor, *F*(3,69) = 1.72, **p* *= .17, η^2^ = 0.07; and social reward, *F*(3,69) = 0.57, **p* *= .64, η^2^ = 0.02. Overall music reward scores also indicated no significant difference, *F*(3,69) = 0.88, **p* *= .46, η^2^ = 0.04. The BMRQ results indicated no significant differences between the patient groups and healthy controls regarding how music engagement was experienced. The absence of significant differences suggests that music engagement experiences do not vary significantly among groups.

### Semantic differential profile scale

**Fig 2:**
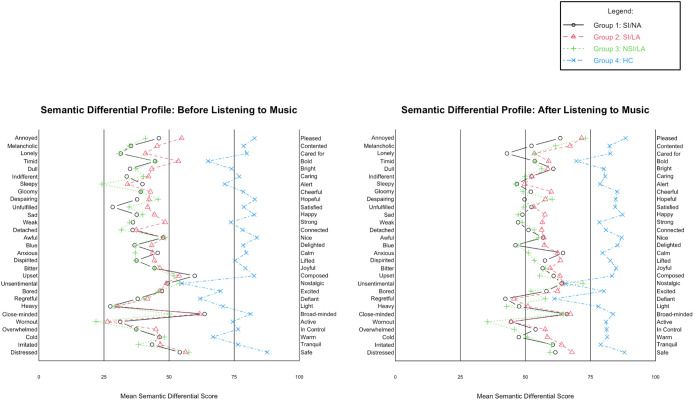
Semantic differential ratings before and after listening to preferred music. *Note.* Patient Groups: SI/NA: suicidal ideation and no attempt (black), SI/LA: suicidal ideation and lifetime attempt (red), NSI/LA: no suicidal ideations and lifetime attempt (green), HC: healthy controls (blue). HC consistently rated self-perception significantly higher than patient groups before and after music listening (**p* *< .001).

The semantic differential profile ratings were evaluated before and after listening to five to seven minutes of preferred music. These bipolar ratings, which range from 0 to 100, were designed to capture a spectrum of responses, with 0 indicating the most negative attitude towards self and 100 the most positive attitude towards self. Descriptive statistics for overall mean ratings for each participant group, before and after listening to preferred music are presented in Table 4. Ratings before and after listening to preferred music are illustrated in Fig 2.

**Table 4:** Descriptive statistics for overall semantic differential ratings.

	Pre	Post
	*M(SD)*	*M(SD)*
Group 1: SI/NA (n = 18)	41.1 (8.6)	53.8 (6.9)
Group 2: SI/LA (n = 12)	44.8 (7.4)	58.6 (6.4)
Group 3: NSI/LA (n = 14)	39.7 (8.3)	54.8 (8.0)
Healthy Controls (n = 28)	76.0 (7.1)	80.9 (6.2)

*Note.* Patient Groups: SI/NA: suicidal ideation and no attempt, SI/LA: suicidal ideation and lifetime attempt, NSI/LA: no suicidal ideations and lifetime attempt.

Before listening to preferred music, the overall mean ratings for all patient groups were below 50 (see Table 4). This suggests that individuals in patient groups were experiencing more negative perception of self, with only six constructs that rated slightly above 50: “annoyed – pleased”, “timid – bold”, “upset – composed”, “unsentimental – nostalgic”, “close-minded – broad-minded”, and “distressed – safe”. This indicates a general negative trend of self-perception among the patient groups prior to music listening. In contrast, the healthy control group exhibited ratings that were consistently above 50, with an overall mean rating of 76.0. This suggests that the healthy controls were experiencing more positive attitudes towards self before engaging with music.

After listening to preferred music, there was a positive shift in attitudes towards self for overall mean ratings for patient groups (see Table 4). Fourteen constructs were rated below 50: “lonely – cared for”, “indifferent – caring”, “sleepy – alert”, “gloomy – cheerful”, “despairing – hopeful”, “unfulfilled – satisfied”, “sad – happy”, “weak-strong”, “blue – delighted”, “regretful – defiant”, “heavy – light”, “worn out – active”, “overwhelmed – in control”, and “cold – warm”. Overall mean ratings for patient groups after listening to music are above 50, but do not meet the average ratings of healthy controls. The healthy control group maintained higher, more positive ratings, with no scores below 60 and an overall mean rating of 80.9, suggesting continued positive attitudes towards self after listening to five to seven minutes of preferred music.

A two-way ANOVA was conducted to examine the effects of participant group and time points (before and after listening to music) on the semantic differential profile scores. The analysis revealed a significant interaction effect between participant group and time points, *F*(3, 224) = 4.32, **p* *= .006, η^2^ = 0.05, indicating that the change in semantic differential profile ratings before and after listening to music significantly differed between groups. Post-hoc analysis using Tukey’s HSD test revealed significant differences between several participant group and time points comparisons (see Table 5).

**Table 5:** Post-hoc comparisons of significant main effects and interactions for participant groups and time points on semantic differential profile scores.

Comparison	Difference (*M*)	95% CI	*p*-value
Group			
HC vs SI/NA	30.36	26.55, 34.17	<.001
SI/LA vs SI/NA	4.21	0.47, 7.95	.02
NSI/LA vs SI/LA	-4.59	-8.40, -0.79	.01
HC vs SI/LA	26.14	22.34, 29.95	<.001
HC vs NSI/LA	30.62	26.88, 34.36	<.001
Time Points			
After vs Before Listening to Music	11.28	9.27, 13.29	<.001
Group: Time			
Before Listening to Music			
HC vs SI/NA	34.22	27.85, 40.59	<.001
HC vs SI/LA	30.42	24.05, 36.78	<.001
HC vs NSI/LA	35.13	29.12, 41.63	<.001
After Listening to Music			
HC vs SI/NA	26.49	20.13, 32.86	<.001
HC vs SI/LA	21.87	15.50, 28.24	<.001
HC vs NSI/LA	26.12	19.87, 32.38	<.001
After vs Before Listening to Music			
SI/NA	12.69	6.43, 18.94	<.001
SI/LA	13.51	7.26, 19.76	<.001
NSI/LA	13.96	7.71, 20.22	<.001

*Note.* Patient Groups: SI/NA: suicidal ideation and no attempt, SI/LA: suicidal ideation and lifetime attempt, NSI/LA: no suicidal ideations and lifetime attempt.

The post-hoc analysis revealed that healthy controls consistently had significantly higher semantic profile scores compared to the patient groups, both before and after listening to music (all **p* *< .001). Additionally, there was a statistically significant increase in semantic differential profile scores for all patient groups from before to after listening to music. However, although significant, this increase represents only a marginally positive shift for patient groups. Furthermore, a significant main effect of group was observed, with SI/NA showing significantly lower scores from SI/LA (**p* *= .02) and NSI/LA showing significantly lower scores from SI/LA (**p* *= .01), suggesting a consistent difference in scores between these groups independent of when they completed the semantic differential profile.

These findings support the hypothesis that self-perception changes following music listening, even among individuals with MDD and suicidality, highlighting its potential as a behavioural marker of anhedonia.

## Discussion

The study aimed to evaluate the music responses using the BMRQ and a semantic differential profile rating scale as behavioural markers of suicidality. Notably, the measures have not been used in this context previously, representing a novel approach to understanding how to evaluate responses to pleasurable experiences like music. This is particularly important for individuals with MDD, who experience anhedonia. The findings provide insights into the sensitivity of these measures in detecting differences in self-perception and music engagement among individuals with MDD with varying levels of suicidal ideation, with and without an attempt history, compared to healthy controls.

The BMRQ results indicated no significant differences across groups for musical seeking, emotion evocation, mood regulation, sensory motor, and social reward factors. Additionally, the overall music reward scores showed no significant differences, indicating that the general experience of music reward is relatively consistent across groups. This suggests that regardless of MDD with or without suicidal ideations and behaviours or healthy controls, individuals experience and engage with music in similar ways. Therefore, the BMRQ may not be sensitive enough to detect differences in music engagement between these groups in this context, particularly if individual variations in emotional or motivational responses to music are not fully captured. The BMRQ has previously been used to explore differences in music reward across general and cross-cultural populations [18,19,24,25], however, its focus on music engagement may limit its sensitivity in detecting subtle nuanced or state-dependent differences in this clinical population.

In contrast, the in-house semantic differential profile responses revealed significant shifts. All patient groups showed marginally positive attitudes toward self, however, these attitudes remained significantly below those of healthy controls (*p* < .001) when listening to their preferred piece of music. This suggests that the semantic differential profile, when used before and after listening to music, may serve as a tool for detecting anhedonic responses. While it has been established that listening to music can be a pleasurable experience [26], the marginally positive shift in self-perception among patient groups suggested an anhedonic response after listening to music.

The significant shift indicates that the semantic differential profile is sensitive enough to detect changes in self-perception from before to after listening to music. Despite exposure to pleasurable stimuli like music, patient groups continue to exhibit anhedonic responses, which is consistent with the findings by Mitterschiffthaler et al. [27], who highlight persistent affective deficits in individuals with MDD. This aligns with our results showing significant differences between healthy controls and all patient groups. Post-hoc analysis revealed that healthy controls consistently had higher semantic differential profile scores compared to the patient groups, both before and after listening to music (all *p* < .001). The healthy controls exhibited positive responses, which differed substantially from those of patient groups. This aligns with findings that individuals with depression have a diminished hedonic capacity, suggesting a persistent deficit in experiencing pleasure [28,29]. Despite the significant increase in semantic differential profile scores for all patient groups after listening to music, the scores did not reach a positive attitude towards self.

Beyond the interaction, a significant main effect of group was found between two patient groups. Group differences were consistent between NSI/LA and SI/LA, regardless of when the semantic differential profile scale was completed, with NSI/LA demonstrating significantly lower scores compared to SI/LA (*p* = .01). Group differences were also consistent between SI/NA and SI/LA, with SI/NA demonstrating significantly lower scores compared to SI/LA (*p* = .02). While the significant interaction shows that listening to music influenced the differences between healthy controls and patient groups, the main effect of group indicates that differences in self-perception remained independent of when the semantic differential profile scale was completed for these patient group comparisons. This suggests distinct differences in self-perception among individuals with a lifetime attempt, with and without suicidal ideation, as well as differences between individuals with suicidal ideations, both with and without a lifetime attempt.

These findings indicate that the semantic differential profile scale is sensitive to detecting differences across groups and from before and after listening to music, particularly at the aggregate level. The distinctiveness of the healthy control group’s responses suggests that the scale is capable of capturing the unique group-level differences.

The method used to classify participant groups in this study has limitations. Suicidal ideation was identified exclusively using item 3 of the HAMD, with individuals assigned to the SI/NA and SI/LA groups requiring a score ≥ 2. As well, most participants in the NSI/LA group scored 1 on this item, indicating mild or passive suicidal thoughts. This approach likely contributed to the misclassification of participants’ suicidal ideation, as single-item measures have been shown to inadequately reflect the complexities. Prior studies have highlighted that reliance on a single question can result in under- or over-identification of suicidality, limiting the accuracy [30,31]. Future research should incorporate multi-item tools to provide a more comprehensive and reliable evaluation of suicidal thoughts and behaviours. Additionally, the study did not account for the time elapsed since participants’ most recent suicide attempt, categorising them solely based on lifetime history. This presents further limitations as responses to the assessments and music-based tasks may be influenced by the recency of suicidal behaviour.

While ANOVAs were used for statistical analysis, the assumption of normality was not met, presenting a limitation given the small sample and unequal group sizes. Although ANOVA is robust to violations of normality, the small sample size increases the risk of reduced statistical power and inflated Type I or II error rates. To address this, nonparametric tests were conducted and yielded consistent findings, suggesting that the ANOVA results were reliable despite these limitations. Nevertheless, the use of parametric tests under these conditions may still affect the precision findings and should be interpreted with caution.

This study relied on self-reported measures, which can be subject to social-desirability or demand bias and could distort the accuracy of participants’ responses. Additionally, the semantic differential profile scale used was developed in-house and has not yet undergone formal psychometric validation, limiting interpretability of results drawn from this measure. Future research should prioritise validating and refining the semantic differential profile scale, which demonstrated greater sensitivity than the BMRQ. These findings support its potential as a tool for evaluating suicidality through music-based responses. Continued investigation of the scale could provide deeper insights into the underlying mechanisms of music’s effect on self-perception and anhedonia. Additionally, investigating the use of these combined measures in different populations at suicide risk could help validate the findings and enhance the applicability of the semantic differential profile in response to music as a tool for evaluating suicide risk.

These findings have several important implications. The lack of significant differences in BMRQ results across groups suggests that individuals with MDD, regardless of suicide ideation or lifetime attempt, and healthy controls engage with and experience music in similar ways. This highlights the universal nature of music as a form of engagement and enjoyment. Salimpoor et al. [32] indicate that the cognitive mechanisms underlying music enjoyment involve predictive processes in the brain, which induce emotional responses and suggest why music is a global phenomenon. The significant shifts in self-perception measured by the semantic differential profile, despite being marginally positive, suggest that this tool is sensitive enough to detect changes in self-perception post-music listening. Additionally, the semantic differential profile may have the ability to detect persistent anhedonic responses suggesting its potential utility in evaluating suicidality.

## Supporting information

S1 File Data for the Barcelona Music Reward Questionnaire (BMRQ) and the semantic differential profile scale. (XLSX)
